# Predictive value of perilesional edema volume in melanoma brain metastasis response to stereotactic radiosurgery

**DOI:** 10.1007/s11060-024-04818-9

**Published:** 2024-09-11

**Authors:** Mariya Yavorska, Miriam Tomaciello, Antonio Sciurti, Elisa Cinelli, Giovanni Rubino, Armando Perrella, Alfonso Cerase, Pierpaolo Pastina, Giovanni Luca Gravina, Silvia Arcieri, Maria Antonietta Mazzei, Giuseppe Migliara, Valentina Baccolini, Francesco Marampon, Giuseppe Minniti, Anna Maria Di Giacomo, Paolo Tini

**Affiliations:** 1 Unit of Radiation Oncology, Department of Medicine, Surgery and Neurosciences, University of Siena, Siena, Italy; 2 Radiation Oncology, Policlinico Umberto I, Department of Radiological, Oncological and Pathological Sciences, Sapienza University of Rome, Rome, Italy; 3 Department of Public Health and Infectious Diseases, Sapienza University of Rome, Rome, Italy; 4 Unit of Neuroradiology, Azienda Ospedaliero-Universitaria Senese, Siena, Italy; 5 Department of Biotechnological and Applied Clinical Sciences, University of L’Aquila, L’Aquila, Italy; 6 Policlinico Umberto I Hospital, Viale del Policlinico, Rome, 00161 Italy; 7 Unit of Diagnostic Imaging, Department of Medicine, Surgery and Neurosciences, University of Siena, Siena, Italy; 8 IRCSS Neuromed, Pozzilli, Italy; 9 Center for Immuno-Oncology, Department of Medicine, Surgery and Neurosciences, University of Siena, Siena, Italy

**Keywords:** Melanoma brain metastasis, Perilesional edema, Radio-surgery, Stereotactic radiotherapy, Immunotherapy

## Abstract

**Background and aim:**

Stereotactic radiotherapy (SRT) is an established treatment for melanoma brain metastases (MBM). Recent evidence suggests that perilesional edema volume (PEV) might compromise the delivery and efficacy of radiotherapy to treat BM. This study investigated the association between SRT efficacy and PEV extent in MBM.

**Materials and methods:**

This retrospective study reviewed medical records from January 2020 to September 2023. Patients with up to 5 measurable MBMs, intracranial disease per RANO/iRANO criteria, and on low-dose corticosteroids were included. MRI scans assessed baseline neuroimaging, with PEV analyzed using 3D Slicer. SRT plans were based on MRI-CT fusion, delivering 18–32.5 Gy in 1–5 fractions. Outcomes included intracranial objective response rate (iORR) and survival measures (L-iPFS and OS). Statistical analysis involved decision tree analysis and multivariable logistic regression, adjusting for clinical and treatment variables.

**Results:**

Seventy-two patients with 101 MBM were analyzed, with a mean age of 68.83 years. The iORR was 61.4%, with Complete Response (CR) in 21.8% and Partial Response (PR) in 39.6% of the treated lesions. PEV correlated with KPS, BRAF status, and treatment response. Decision tree analysis identified a PEV cutoff at 0.5 cc, with lower PEVs predicting better responses (AUC = 0.82 sensitivity: 86.7%, specificity:74.4%,). Patients with PEV ≥ 0.5 cc had lower response rates (iORR 44.7% vs. 63.8%, *p* < 0.001). Median OS was 9.4 months, with L-iPFS of 27 months. PEV significantly impacted survival outcomes.

**Conclusions:**

A more extensive PEV was associated with a less favorable outcome to SRT in MBM.

## Introduction

Brain metastases from melanoma represent a significant clinical challenge due to their poor prognosis and the complexities of treatment. Melanoma, known for its aggressive nature, frequently metastasizes to the brain, complicating management and impacting survival outcomes [1]. The incidence of brain metastases in melanoma patients has increased with advances in systemic therapies, highlighting the need for effective local treatment strategies [2]. Despite improvements in targeted therapies and immunotherapies, patients with melanoma brain metastases often face limited treatment options and a diminished quality of life [3]. Radiotherapy (RT), whether used alone or in conjunction with surgery and/or systemic therapy, remains a key treatment strategy for managing BMs [4]. Specifically, stereotactic radiotherapy (SRT) is employed to treat patients with up to four unresected BMs, each with a diameter of 30 mm or less, as well as the surgical cavities of patients who have had one or two BMs removed [4–7]. SRT achieves local control rates ranging from 75 to 95% [8–10], leads to a better quality of life (QoL) compared to whole brain radiation therapy (WBRT) alone [3–4], and can work in synergy with systemic therapies, including immune checkpoint inhibitors (ICIs) [11–15]. Despite these benefits, the prognosis for BMs treated with SRT remains poor, with median overall survival (OS) being less than one year [16, 17]. To date, only a few predictive factors for response have been identified, such as the Karnofsky performance score (KPS), the number of BMs, presence of extracranial metastases, certain molecular and radiomic characteristics, the dose/volume ratio, and the concurrent use of systemic therapies, among others [18–21].

Perilesional edema volume (PEV) is a significant cause of morbidity in patients with both primary and metastatic brain tumors [22]. It has been associated with cancer cell infiltration [23, 24], hypoxia, and neovascularization [25], all of which are known to hinder the effectiveness of radiation and systemic therapies. Larger PE diameters have been linked to a higher risk of intracranial progression and a reduced likelihood of responding to SRT [26–28] or systemic treatments [29] for BMs originating from NSCLC. However, the role of PEV as a predictive factor for response to SRT in brain metastases from melanoma (MBM) remains unclear.

This study seeks to assess the impact of PEV on intracranial response and its association with survival in patients with MBM treated with SRT in combination with systemic therapy.

## Methods

### Patients selection

This retrospective study was performed at the Radiation Oncology Unit of Azienda Ospedaliera Universitaria Senese in Siena, Italy, covering the period from January 2020 to September 2023. Clinical characteristics, histopathological findings, molecular profiles, and details of systemic treatments were gathered from patient medical records. The inclusion criteria for this study included: (i) patients with up to 5 melanoma brain metastases (MBMs); (ii) measurable intracranial disease according to RANO [30] and iRANO [31] guidelines; (iii) treatment involving stereotactic radiotherapy (SRT); and (iv) administration of a low dose of corticosteroids (less than 2 mg/day of dexamethasone) at the time of the brain MRI prior to SRT. Exclusion criteria involved: (i) any prior treatment for MBMs; (ii) prior surgical removal or whole-brain radiation therapy (WBRT); (iii) diagnosis of meningeal carcinomatosis; and (iv) absence of a baseline brain MRI. The study was conducted following the principles of the Declaration of Helsinki and received ethical approval from the institutional review board of “Le Scotte” Hospital of Siena. Written informed consent was obtained from each participant, and patient confidentiality was maintained by anonymizing all data prior to analysis.

### Imaging and measurements

**Fig. 1:**
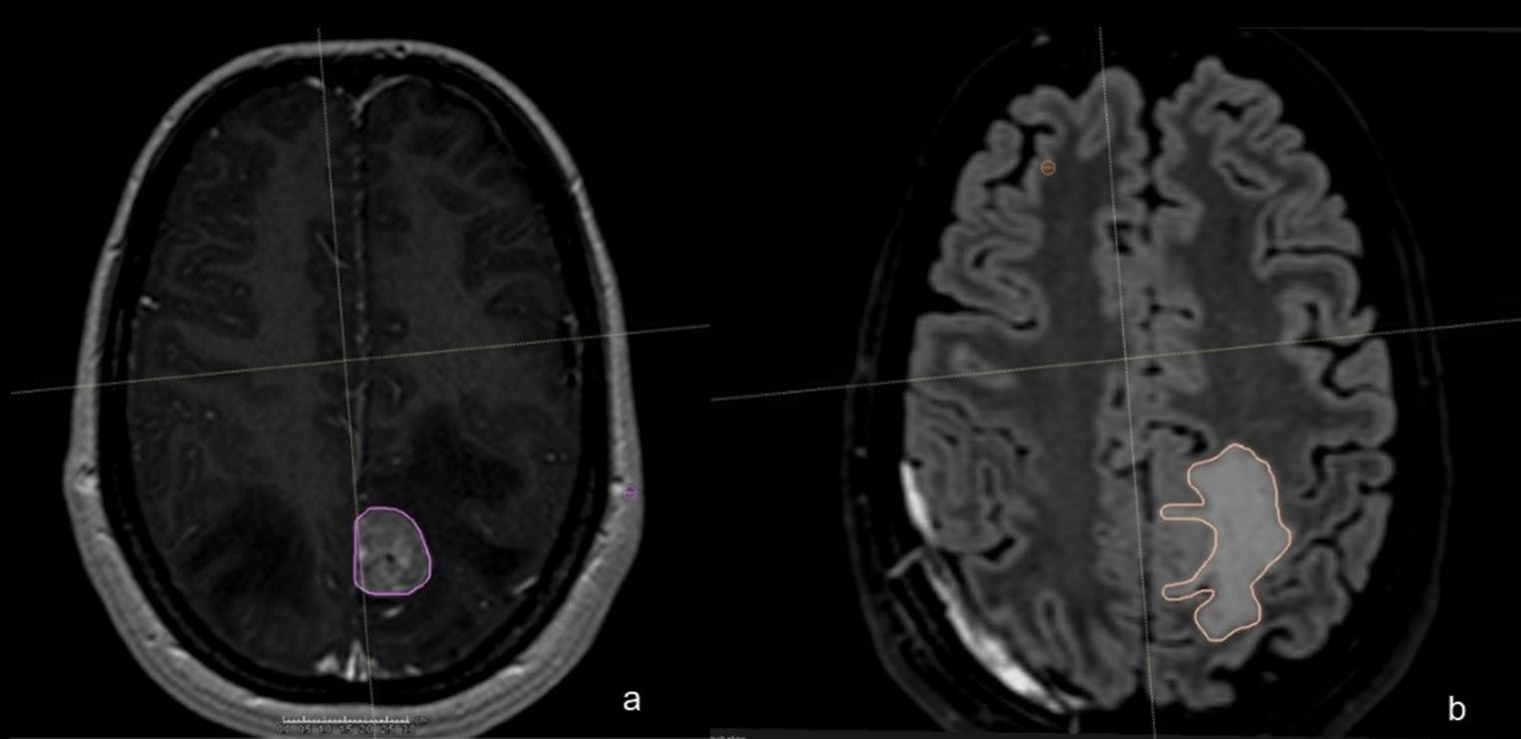
Segmentation of Gross Tumor Volume (GT) on contrast-enhanced T1-weighted images (a) and Perilesional Edema Volume (PEV) on FLAIR-weighted images

**Fig. 2:**
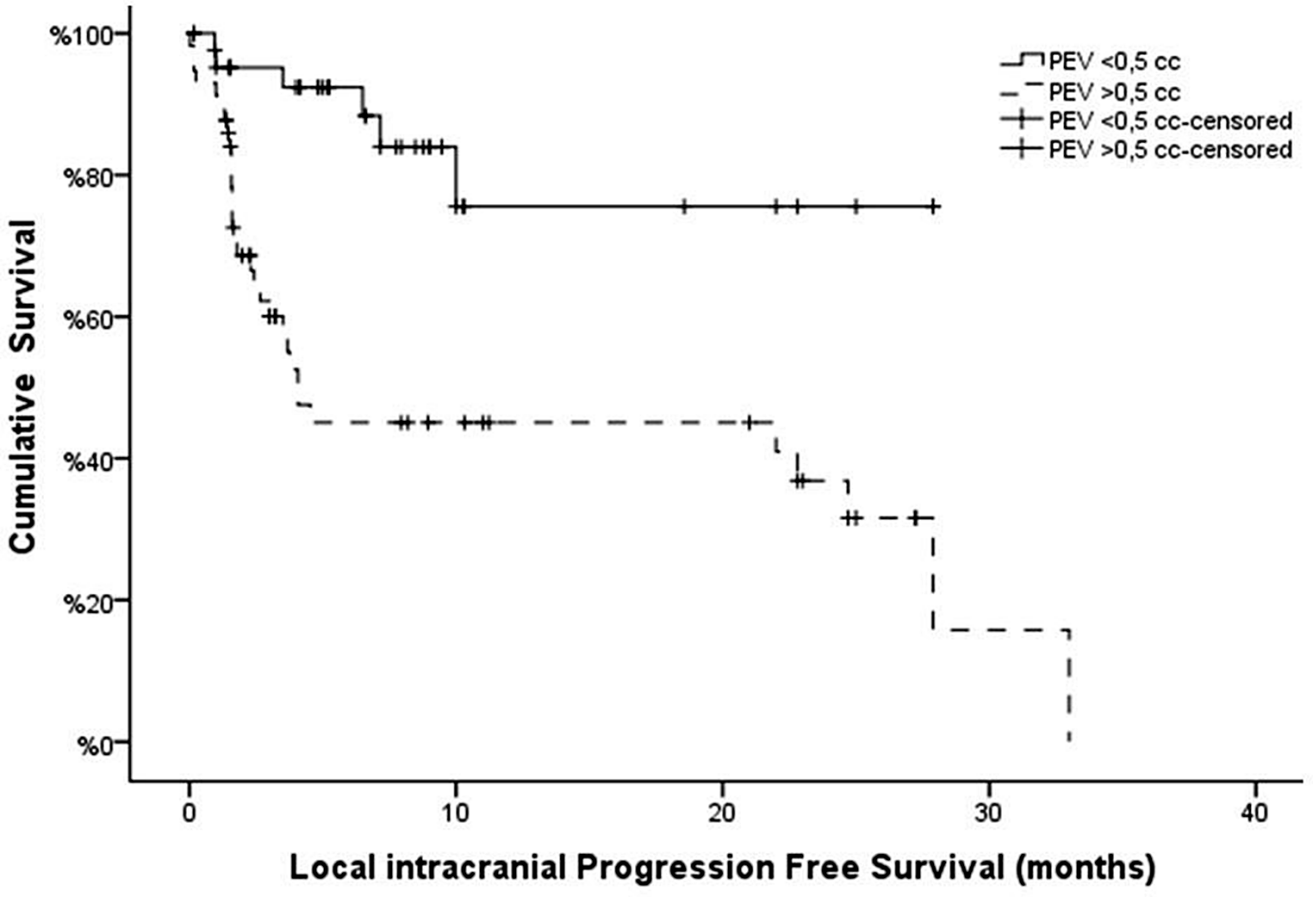
Kaplan-Meier curves of Local intracranial Progression Free Survival according Perilesional Edema Volume (PEV) with a cut-off value of 0,5 cc (*p* = 0,001)

This study exclusively utilized MRI scans for imaging assessments. Standard MRI sequences included axial T1, T2-weighted, and FLAIR images. Baseline neuroimaging features were independently evaluated by two radiation oncologists and a neuroradiologist before the initiation of local therapy. The MRIs obtained at enrollment were analyzed using 3D Slicer software (https://www.slicer.org*).* For each MBM, segmentation was performed on contrast-enhanced 3D T1-weighted images to determine the gross tumor (GT) volume (Fig. 1a). The volume of perilesional edema (PEV) was quantified by segmenting FLAIR/T2-weighted images (Fig. 1b). The Fast GrowCut Extension with Laplacian 0 settings was used to create 3D models. Both PE and GT volumes were measured in cubic centimeters (cc). Tumors that exhibited overlapping edema due to proximity to other lesions or were incompatible with 3D Slicer’s processing were excluded from the analysis (See Fig. 2).

### SRT and systemic treatments

Treatment plans were developed by integrating thin-slice MRI with stereotactic CT scans. The gross tumor volume (GTV) was delineated as the entire visible lesion on the CT/MRI fusion. To account for potential errors in imaging fusion, contouring, setup variations, and patient movement during treatment, a 3 mm isotropic margin was added to the GTV to form the planning target volume (PTV). SRT was administered with a total dose of 18–32.5 Gy delivered over 1 to 5 fractions. The total dose, fractionation schedule, and concomitant systemic therapies were individualized based on discussions in a multidisciplinary tumor board.

### Outcome measures

The primary outcome of interest was the intracranial objective response rate (iORR), defined as the percentage of patients achieving either a complete response (CR) or partial response (PR) according RANO criteria. Brain contrast-enhanced MRI was conducted at baseline, 8 to 10 weeks post-SRT, and subsequently every 4 to 6 months or as clinically indicated. The duration of intracranial response (L-iPFS) was measured from the time of SRT to the occurrence of local intracranial progression. Overall survival (OS) was defined as the period from RT to death from any cause. To ensure the accuracy of response evaluations, they were independently reassessed by both a radiation oncologist and a neuroradiologist.

### Statistical analysis

Continuous variables were summarized using medians and interquartile ranges, while categorical variables were presented as frequencies and percentages. A decision tree analysis was performed to determine the cut-off point for PEV that predicted treatment response. The analysis included: Selection of ‘Response’ as the dependent variable and ‘Edema_Volume’ as the independent variable; application of the Classification and Regression Trees (C&RT) method for its interpretability and ability to manage non-linear relationships; setting the maximum tree depth to 1 to establish a single cut-off point, and using the Gini impurity measure for splitting; validation through a 10-fold cross-validation approach; and examination of the resulting decision tree structure and classification rules. Comparisons between patient groups classified by PEV cut-off points were conducted using the Mann-Whitney U-test for continuous variables and the χ2 test for categorical variables. Based on expert input [31], a multivariable logistic regression model was created to evaluate the impact of PEV on treatment response, adjusting for gender, age, gross tumor volume, SRT dose (Gy) per fraction, and type of systemic therapy (none, immune checkpoint inhibitors (ICI), targeted therapy (TT), or a combination of ICI and TT). Adjusted Odds Ratios (aORs) for PR or CR and their 95% confidence intervals were calculated. All statistical analyses were performed using IBM SPSS Statistics (version 20.0), with significance defined as a two-sided p-value < 0.05.

## Results

### Patients’ characteristics

Seventy-two patients with confirmed diagnoses of MBM met the inclusion criteria and were eligible for analysis. The mean age was 68.83 years (IQR: 61.0–77.0), with 59.3% being male. At diagnosis, 20 patients had a Karnofsky Performance Status (KPS) < 80, and 72.8% (*n* = 52) had multiple MBM. A total of 101 MBMs were treated with SRT (Table 1). The mean total prescription dose was 24.57 Gy (range: 14–32.5 Gy), with a median of 27 Gy; 9 Gy per fraction was the most common dose. Seventy-three MBMs (72.3%) received the total dose in 3 fractions, 10 (9.9%) in a single fraction, and 18 (17.8%) in 5 fractions. SRT was performed concurrently with immune checkpoint inhibitors (ICI) in 56.4% (*n* = 56) of treated MBM, with targeted therapy (TT) in 11.9% (*n* = 12), and without concurrent systemic treatment in 31.7% (*n* = 32).

**Table 1:** Characteristics of Melanoma Brain Metastasis (*N* = 101) by Perilesional Edema volume (cm3)

	Total	Perilesion Edema < 0,5 cm ^3^	Perilesion Edema > 0,5 cm ^3^	*p*-value
Patients, N	72			
MBM, N	101	45	56	
Age, Median (IQR)	69,0 (61,0–77,0)	69,0 (63,0–78,0)	69,5 (60,0–76,0)	0,914
Gender, N(%) Female Male	41 (40,6) 60 (59,4)	26 (58,1) 19 (41,9)	15 (26,3) 41 (73,7)	0,008
BRAF mutational status, N (%) Wild-type Mutated Unknown	46 (45,5) 51 (50,5) 4 (4)	17 (16,8) 25 (24,8) 1 (1)	29 (28,7) 26 (25,7) 3 (3)	0,086
Total RT dose (Gy), Median (IQR)	27,0 (24,0–27,0)	27,0 (24,0–30,0)	27,0 (21,0–30,0)	0,965
N° of RT fractions, N (%) 1 3 5	10 (9,9) 73 (72,3) 18 (17,8)	4 (3,9) 32 (31,7) 2 (1,9)	6 (5,9) 41 (40,6) 16 (15,9)	0,250
Systemic therapy, N (%) None Immunotherapy (IT) Target therapy ± IT	32 (31,7) 57 (56,4) 12 (11,9)	20 (19,8) 19 (18,8) 5 (5)	12 (11,9) 38 (37,6) 7 (6,9)	0,067
Gross tumor volume (cm ^3^), Median (IQR)	0,7 (0,2–2,3)	0,3 (0,1 − 0,4)	2,0 (0,9 − 3,5)	< 0,001
Treatment Response, N (%) CR PR SD PD	22 (21,8) 40 (39,6) 21 (20,8) 18 (17,8)	20 (19,8) 23 (22,8) 1 (1) 1 (1)	2 (2) 17 (16,8) 20 (19,8) 17 (16,8)	< 0,001

MBM: Melanoma Brain Metastasis; RT: Radiotherapy; CR: Complete Response; PR: Partial Response; SD: Stable Disease; PD: Progression Disease

### Treatment outcomes

Post-SRT, complete response (CR) was observed in 21.8% (*n* = 22) of treated MBMs, partial response (PR) in 39.6% (*n* = 40), yielding an intracranial objective response rate (iORR) of 61.4%. Treated lesions had a mean gross tumor (GT) volume of 1.41 cc (median: 0.7 cc) and a mean perilesional edema (PEV) volume of 3.6 cc (median: 1.9 cc). PEV correlated significantly with KPS (*p* < 0.001), BRAF mutation status (*p* = 0.022), GT volume (*p* < 0.001), and iORR (CR + PR) (*p* < 0.001).

### Decision Tree Analysis

A decision tree analysis identified a PEV cutoff of 0.5 cc related to iORR. Patients with PEV ≤ 0.5 cc were more likely to respond to treatment compared to those with volumes > 0.5 cc (sensitivity: 86.7%, specificity: 74.4%, AUC = 0.82 [0.67–0.95]). iORR (CR + PR) was achieved in 95.5% of patients with PE volume ≤ 0.5 cc, compared to 33.9% with PEV > 0.5 cc (*p* < 0.001) (Table 1).

### Multivariable analysis

Multivariable analysis (Table 2) showed that a PEV > 0.5 cc was independently associated with a reduced probability of achieving PR or CR (aOR: 0.06, 95% CI: 0.01–0.51), along with higher RT doses (aOR: 1.40, 95% CI: 1.04–1.88) and GT volume (aOR: 0.85, 95% CI: 0.04–0.86). Gender, age, and systemic therapy were not significantly associated with outcomes.

**Table 2:** Logistic regression model for Objective Response (PR or CR) (*N* = 101)

Odds Ratio		95% CI	*p*- value
Perilesional Edema Volume (cm3)			
< 0.5 cm3	Ref.		
≥ 0.5 cm3	0.06	0.01– 0.51	0.010
Gender			
Female	Ref.		
Male	2.74	0.62–12.03	0.182
Age	0.99	0.95– 1.04	0.756
Gross Tumour Volume (cm3)			
< 0.7 cm3	Ref.		
≥ 0.7 cm3	0.85	0.04– 0.86	0.032
RT dose (Gy) per fraction	1.40	1.04– 1.88	0.026
Systemic therapy			
None	Ref.		
Immunotherapy only	1.02	0.21– 4.89	0.976
Targeted therapy and/or immunotherapy	1.93	0.16–23.25	0.605

PR: Partial Response; CR: Complete Response; RT: Radiotherapy

### Survival outcomes

The median overall survival (OS) for the entire cohort was 9.4 months. The median local intracranial progression-free survival (L-iPFS) was 24.7 months, with a 6-month local control rate of 81.0%. Patients with PEV > 0.5 cc had a higher mortality rate compared to those with PEV ≤ 0.5 cc (76.3% vs. 48.4%, *p* = 0.016). Patients with PEV ≤ 0.5 cc had 90% disease control at 6 months and a median L-iPFS not reached, compared to those with PEV > 0.5 cc (*p* = 0.031). Multivariable analysis (Cox regression) showed that L-iPFS was related to PEV (HR: 1.8, 95% CI: 1.2–2.2, *p* = 0.001) but not GT volume (HR: 1.06, 95% CI: 0.5–2.4 *p* > 0.05) (Table 3).

**Table 3:** Results of Cox Regression Analysis for Long-Term Intracranial progression-free survival (L-iPFS) in Melanoma Brain metastases

Variable	Hazard Ratio (HR)	95% CI for HR	*p*-value
PEV (Perilesional Edema Volume**)**	1.85	1.2–2.2	0.005
Dose	1.13	0.82–1.53	0.440
GT Volume	1.06	0.5–2.4	0.620
KPS	1.04	0.93–2.19	0.710
Age	1.0	0.9–1.1	0.390
Systemic Therapy	0.9	0.6–1.2	0.470

**Fig. 3:**
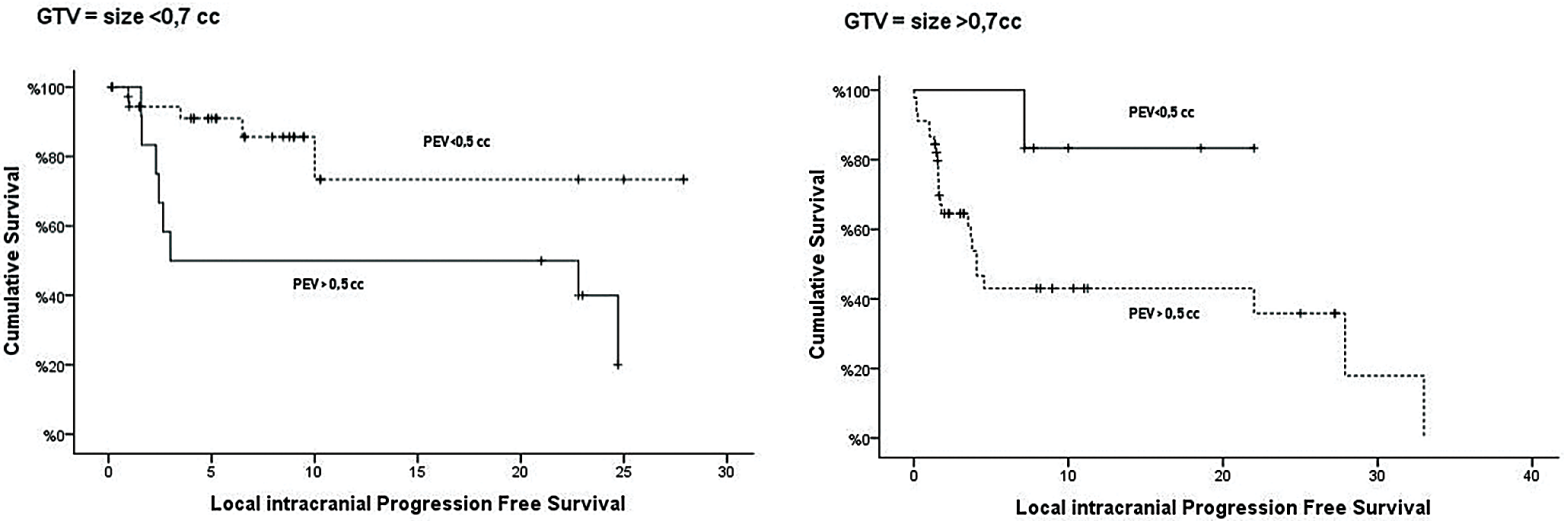
Kaplan-Meier curves of Local intracranial Progression Free Survival according Perilesional Edema Volume (PEV) with a cut-off value of 0,5 cc and Gross Tumor Volume (GTV) with a cut-off value of 0,7 cc

We conducted a subgroup analysis based on the GT volume to further evaluate the prognostic significance of the perilesional edema volume (PEV). The analysis was stratified into two groups: lesions with a volume < 0.7 cc and those > 0.7 cc. The PEV threshold of 0.5 cc was found to be statistically significant in both subgroups. In patients with lesions < 0.7 cc, those with a PEV < 0.5 cc did not reach the median L-iPFS, whereas patients with a PEV > 0.5 cc had a median L-iPFS of 3 months (*p* = 0.01). Similarly, in patients with lesions > 0.7 cc, those with a PEV < 0.5 cc did not reach the median L-iPFS, while patients with a PEV > 0.5 cc had a median L-iPFS of 4 months (*p* = 0.032) (Fig. 3).

OS was associated with PEV (*p* = 0.042, HR: 1.4, 95% CI: 1.01–1.82) and the presence of extracranial disease (*p* = 0.005, HR: 4.3, 95% CI: 2.2–5.2).

## Discussion

This study highlights the relevance of perilesional edema (PE) as a potential biomarker of intracranial response to stereotactic radiotherapy (SRT) in patients with melanoma brain metastases (MBM). PE is a significant cause of morbidity and mortality in patients with central nervous system (CNS) malignancies, including metastases [32]. It is associated with blood-brain barrier disruption, plasma leakage, and impaired oxygen delivery, contributing to a hypoxic tumor microenvironment—a key factor in hypoxia-mediated radioresistance [28]. Hypoxia-inducible factors (HIFs) regulate genes involved in cell survival, glycolysis, angiogenesis, and growth factor expression, all of which promote tumor growth and resistance to radiation therapy (RT) [33]. Additionally, the hypoxic microenvironment leads to genomic instability, reduced DNA repair, and increased cancer stem cell (CSC) activity, further contributing to radioresistance [34, 35].

Our results demonstrate that MBM lesions with lower PE volumes have better complete and partial response (iORR) rates to SRT compared to those with stable or progressive disease, underscoring the negative predictive role of PE. Notably, lesions with minimal or no PEV (< 0.5 cc) exhibit improved local intracranial progression-free survival (L-iPFS) and sustained responses. Multivariate logistic regression analysis, adjusting for potential confounders such as tumor volume and clinical variables, confirmed that PEV is an independent predictor of poor radiological response to SRT (*p* < 0.05). This suggests that the impact of PEV on treatment outcomes is not merely a reflection of tumor size but represents an independent effect likely related to hypoxia, tumor microenvironment changes, and radioresistance associated with extensive PEV.

PEV also emerged as the sole independent predictor of L-iPFS (*p* < 0.01), with minimal or no PEV significantly associated with prolonged L-iPFS. Subgroup analysis based on lesion size (volume < 0.7 cc and > 0.7 cc) reinforced the robustness of these findings across different tumor volumes. These results suggest that PEV could serve as a simple yet robust biomarker for predicting radiosensitivity and guiding personalized treatment strategies.

Moreover, integrating PEV into prognostic models could enhance the accuracy of response predictions and help identify high-risk patients who may benefit from intensified or combination treatments. The significant association between PE volume and overall survival (OS) further underscores the importance of intracranial tumor control for survival, although OS remains heavily influenced by the presence of extracranial disease (HR 4.3). Measuring PE is a simple and accessible method to predict intracranial response following RT and could be integrated into clinical practice for identifying high-risk patients and supporting the rationale for combining anti-angiogenic agents with RT to reduce peritumoral vasogenic edema and improve outcomes.

Agents targeting the VEGF pathway have shown potential in preclinical and clinical models to normalize tumor vasculature, reduce edema, enhance tissue oxygenation, and improve the efficacy of RT, chemotherapy, or immunotherapy [37, 38]. Vascular normalization facilitates the transport of therapeutic agents, enhances radiation-induced DNA damage, activates immune responses, and reduces steroid use, making it easier to implement immune checkpoint inhibitors [39]. Our survival analysis further confirms the prognostic significance of PE, suggesting that incorporating PE into prognostic models could improve accuracy for patients with melanoma-related brain metastases.

The limitations of this study include its retrospective design, small sample size, and the heterogeneity of the patient population, which included various oncogene-driven therapies, limiting the generalizability of the findings.

## Conclusions

Although limited by the small number of patients and the retrospective nature, our study seems to be the first to systematically evaluate the role of PE in response to SRT in melanoma brain metastases, suggesting that PE is a strong predictor of response to RT treatment in melanoma ME. The identification of PE can help to better tailor therapeutic strategies in this context and to identify candidates for treatment intensification strategies, to increase the intracranial response. PE could be a good tool to predict patient survival. It would be useful to investigate the addition of PE to establish prognostic assessment models, further studies are needed to validate these findings. The potential efficacy of anti-angiogenic factors in high-risk patients needs further investigation through phase III controlled trials.

## Data Availability

No datasets were generated or analysed during the current study.

## References

[1] Gritsko VM et al (2009) Brain metastases in melanoma patients: prevalence, management, and outcomes. Cancer. 115(23):5353–5361

[2] Dai C et al (2015) Increasing incidence of brain metastases in melanoma patients: implications for treatment strategies. Melanoma Research. 25(2):125–130

[3] Lin JJ et al (2021) Brain metastases in melanoma: current management and emerging therapies. Curr Oncol Rep 23(7):1–11

[4] Vogelbaum MA, Brown PD, Messersmith H, Brastianos PK, Burri S, Cahill D, Dunn IF, Gaspar LE, Gatson NTN, Gondi V, Jordan JT, Lassman AB, Maues J, Mohile N, Redjal N, Stevens G, Sulman E, van den Bent M, Wallace HJ, Weinberg JS, Zadeh G, Schiff D (2022) Treatment for Brain Metastases: ASCO-SNO-ASTRO Guideline. J Clin Oncol.;40(5):492–516. 10.1200/JCO.21.02314. Epub 2021 Dec 21. Erratum in: J Clin Oncol. 2022;40(12):1392. doi: 10.1200/JCO.22.00593. PMID: 34932393 10.1200/JCO.21.02314 34932393

[5] Minniti G, Clarke E, Lanzetta G, Osti MF, Trasimeni G, Bozzao A, Romano A, Enrici RM (2011) Stereotactic radiosurgery for brain metastases: analysis of outcome and risk of brain radionecrosis. Radiat Oncol 6:48. 10.1186/1748-717X-6-48PMID: 21575163; PMCID: PMC3108308 21575163 10.1186/1748-717X-6-48 PMC3108308

[6] Minniti G, Amelio D, Amichetti M, Salvati M, Muni R, Bozzao A, Lanzetta G, Scarpino S, Arcella A, Enrici RM (2010) Patterns of failure and comparison of different target volume delineations in patients with glioblastoma treated with conformal radiotherapy plus concomitant and adjuvant temozolomide. Radiother Oncol 97(3):377–381 Epub 2010 Sep 18. PMID: 20855119 20855119 10.1016/j.radonc.2010.08.020

[7] Sayan M, Zoto Mustafayev T, Sahin B, Kefelioglu ESS, Wang SJ, Kurup V, Balmuk A, Gungor G, Ohri N, Weiner J, Ozyar E, Atalar B (2019) Evaluation of response to stereotactic radiosurgery in patients with radioresistant brain metastases. Radiat Oncol J 37(4):265–270 Epub 2019 Dec 31. PMID: 31918464; PMCID: PMC6952719 31918464 10.3857/roj.2019.00409 PMC6952719

[8] Petrelli F, De Stefani A, Trevisan F, Parati C, Inno A, Merelli B, Ghidini M, Bruschieri L, Vitali E, Cabiddu M, Borgonovo K, Ghilardi M, Barni S, Ghidini A (2019) Combination of radiotherapy and immunotherapy for brain metastases: a systematic review and meta-analysis. Crit Rev Oncol Hematol 144:102830 Epub 2019 Nov 1. PMID: 31733443 31733443 10.1016/j.critrevonc.2019.102830

[9] Lehrer EJ, Peterson J, Brown PD, Sheehan JP, Quiñones-Hinojosa A, Zaorsky NG, Trifiletti DM (2019) Treatment of brain metastases with stereotactic radiosurgery and immune checkpoint inhibitors: an international meta-analysis of individual patient data. Radiother Oncol 130:104–112 Epub 2018 Sep 18. PMID: 30241791 30241791 10.1016/j.radonc.2018.08.025

[10] Brown PD, Jaeckle K, Ballman KV, Farace E, Cerhan JH, Anderson SK, Carrero XW, Barker FG 2, Deming R, Burri SH, Ménard C, Chung C, Stieber VW, Pollock BE, Galanis E, Buckner JC, Asher AL (2016). Effect of Radiosurgery Alone vs Radiosurgery With Whole Brain Radiation Therapy on Cognitive Function in Patients With 1 to 3 Brain Metastases: A Randomized Clinical Trial. JAMA. doi: 10.1001/jama.2016.9839. Erratum in: JAMA. 2018;320(5):510. doi: 10.1001/jama.2018.9890. PMID: 27458945; PMCID: PMC5313044. 10.1001/jama.2016.9839 PMC5313044 27458945

[11] Moraes FY, Taunk NK, Marta GN, Suh JH, Yamada Y (2016) The rationale for targeted therapies and stereotactic radiosurgery in the treatment of Brain metastases. Oncologist 21(2):244–251. 10.1634/theoncologist.2015-0293Epub 2016 Jan 13. PMID: 26764249; PMCID: PMC4746085 26764249 10.1634/theoncologist.2015-0293 PMC4746085

[12] He Q, Zhang C, Tang S, Li J, Ren Q (2020;) Intracranial radiotherapy with or without immune checkpoint inhibition for brain metastases: a systematic review and meta-analysis. Transl Cancer Res 9(10):5909–5924. 10.21037/tcr-20-902. PMID: 35117204; PMCID: PMC8797322 10.21037/tcr-20-902 PMC8797322 35117204

[13] Kim JH, Jenrow KA, Brown SL (2018) Novel biological strategies to enhance the radiation therapeutic ratio. Radiat Oncol J 36(3):172–181. 10.3857/roj.2018.00332Epub 2018 Sep 30. PMID: 30309208; PMCID: PMC6226138 30309208 10.3857/roj.2018.00332 PMC6226138

[14] Marampon F, Gelibter AJ, Cicco PR, Parisi M, Serpone M, De Felice F, Bulzonetti N, Musio D, Cortesi E, Tombolini V (2022) Safety and efficacy of combining afatinib and whole-brain radiation therapy in treating brain metastases from EGFR-mutated NSCLC: a case report and literature review. BJR Case Rep 8(5):20200134. 10.1259/bjrcr.20200134PMID: 36211614; PMCID: PMC9518736 36211614 10.1259/bjrcr.20200134 PMC9518736

[15] Guénolé M, Lucia F, Bourbonne V, Dissaux G, Reygagne E, Goasduff G, Pradier O, Schick U (2020) Impact of concomitant systemic treatments on toxicity and intracerebral response after stereotactic radiotherapy for brain metastases. BMC Cancer 20(1):991. 10.1186/s12885-020-07491-zPMID: 33050910; PMCID: PMC7557085 33050910 10.1186/s12885-020-07491-z PMC7557085

[16] Brown PD, Jaeckle K, Ballman KV, Farace E, Cerhan JH, Anderson SK, Carrero XW, Barker FG 2nd, Deming R, Burri SH, Ménard C, Chung C, Stieber VW, Pollock BE, Galanis E, Buckner JC, Asher AL (2016) Effect of Radiosurgery Alone vs Radiosurgery With Whole Brain Radiation Therapy on Cognitive Function in Patients With 1 to 3 Brain Metastases: A Randomized Clinical Trial. JAMA.;316(4):401–409. 10.1001/jama.2016.9839. Erratum in: JAMA. 2018;320(5):510. doi: 10.1001/jama.2018.9890. PMID: 27458945; PMCID: PMC5313044 10.1001/jama.2016.9839 PMC5313044 27458945

[17] Brown PD, Ballman KV, Cerhan JH, Anderson SK, Carrero XW, Whitton AC, Greenspoon J, Parney IF, Laack NNI, Ashman JB, Bahary JP, Hadjipanayis CG, Urbanic JJ, Barker FG 2nd, Farace E, Khuntia D, Giannini C, Buckner JC, Galanis E, Roberge D (2017) Postoperative stereotactic radiosurgery compared with whole brain radiotherapy for resected metastatic brain disease (NCCTG N107C/CEC·3): a multicentre, randomised, controlled, phase 3 trial. Lancet Oncol.;18(8):1049–1060. doi: 10.1016/S1470-2045(17)30441-2. Epub 2017 Jul 4. PMID: 28687377; PMCID: PMC5568757 10.1016/S1470-2045(17)30441-2 PMC5568757 28687377

[18] Simonsen MK, Vrou Offersen B, Jensen AB (2023) Prognosis of breast cancer patients with brain metastasis treated with radiotherapy. Acta Oncol 62(8):871–879 Epub 2023 Jul 27. PMID: 37498539 37498539 10.1080/0284186X.2023.2238551

[19] Franceschini D, De Rose F, Franzese C, Comito T, Di Brina L, Radicioni G, Evangelista A, D’Agostino GR, Navarria P, Scorsetti M (2019) Predictive factors for response and survival in a cohort of oligometastatic patients treated with stereotactic body Radiation Therapy. Int J Radiat Oncol Biol Phys 104(1):111–121 Epub 2019 Jan 8. PMID: 30630030 30630030 10.1016/j.ijrobp.2018.12.049

[20] Amsbaugh MJ, Yusuf MB, Gaskins J, Dragun AE, Dunlap N, Guan T, Woo SA, Dose-Volume (2017) Response model for brain metastases treated with Frameless single-fraction robotic radiosurgery: seeking to Better Predict response to treatment. Technol Cancer Res Treat 16(3):344–351 Epub 2016 Dec 27. PMID: 28027696; PMCID: PMC5616050 10.1177/1533034616685025 PMC5616050 28027696

[21] Jaberipour M, Soliman H, Sahgal A, Sadeghi-Naini A (2021) A priori prediction of local failure in brain metastasis after hypo-fractionated stereotactic radiotherapy using quantitative MRI and machine learning. Sci Rep 11(1):21620. 10.1038/s41598-021-01024-9PMID: 34732781; PMCID: PMC8566533 34732781 10.1038/s41598-021-01024-9 PMC8566533

[22] Gavrilovic IT, Posner JB (2005) Brain metastases: epidemiology and pathophysiology. J Neurooncol.;75(1):5–14. 10.1007/s11060-004-8093-6. PMID: 16215811 10.1007/s11060-004-8093-6 16215811

[23] Kerschbaumer J, Bauer M, Popovscaia M, Grams AE, Thomé C, Freyschlag CF (2017) Correlation of Tumor and Peritumoral Edema Volumes with Survival in Patients with Cerebral Metastases. Anticancer Res.;37(2):871–875. 10.21873/anticanres.11392. PMID: 28179345 10.21873/anticanres.11392 28179345

[24] Chang EL, Akyurek S, Avalos T, Rebueno N, Spicer C, Garcia J, Famiglietti R, Allen PK, Chao KS, Mahajan A, Woo SY, Maor MH (2007) Evaluation of peritumoral edema in the delineation of radiotherapy clinical target volumes for glioblastoma. Int J Radiat Oncol Biol Phys.;68(1):144– 50. doi: 10.1016/j.ijrobp.2006.12.009. Epub 2007 Feb 15. PMID: 17306935 10.1016/j.ijrobp.2006.12.009 17306935

[25] Spanberger T, Berghoff AS, Dinhof C, Ilhan-Mutlu A, Magerle M, Hutterer M, Pichler J, Wöhrer A, Hackl M, Widhalm G, Hainfellner JA, Dieckmann K, Marosi C, Birner P, Prayer D, Preusser M (2013) Extent of peritumoral brain edema correlates with prognosis, tumoral growth pattern, HIF1a expression and angiogenic activity in patients with single brain metastases. Clin Exp Metastasis 30(4):357–368. 10.1007/s10585-012-9542-9Epub 2012 Oct 17. PMID: 23076770 23076770 10.1007/s10585-012-9542-9

[26] Tini P, Nardone V, Pastina P, Battaglia G, Vinciguerra C, Carfagno T, Rubino G, Carbone SF, Sebaste L, Cerase A, Federico A, Pirtoli L (2017) Perilesional edema in brain metastasis from non-small cell lung cancer (NSCLC) as predictor of response to radiosurgery (SRS). Neurol Sci.;38(6):975–982. 10.1007/s10072-017-2876-y. Epub 2017 Mar 4. PMID: 28260188 10.1007/s10072-017-2876-y 28260188

[27] Nardone V, Nanni S, Pastina P, Vinciguerra C, Cerase A, Correale P, Guida C, Giordano A, Tini P, Reginelli A, Cappabianca S, Pirtoli L (2019) Role of perilesional edema and tumor volume in the prognosis of non-small cell lung cancer (NSCLC) undergoing radiosurgery (SRS) for brain metastases. Strahlenther Onkol 195(8):734–744 English. doi:. 10.1007/s00066-019-01475-0 31123785 10.1007/s00066-019-01475-0

[28] Arrieta O, Bolaño-Guerra LM, Caballé-Pérez E, Lara-Mejía L, Turcott JG, Gutiérrez S, Lozano-Ruiz F, Cabrera-Miranda L, Arroyave-Ramírez AM, Maldonado-Magos F, Corrales L, Martín C, Gómez-García AP, Cacho-Díaz B, Cardona AF (2023) Perilesional edema diameter associated with brain metastases as a predictive factor of response to radiotherapy in non-small cell lung cancer. Front Oncol 13:1251620. 10.3389/fonc.2023.1251620PMID: 37916162; PMCID: PMC10616784 37916162 10.3389/fonc.2023.1251620 PMC10616784

[29] Alemany M, Domènech M, Argyriou AA, Vilariño N, Majós C, Naval-Baudin P, Lucas A, Palmero R, Simó M, Nadal E, Bruna J (2021) Perilesional edema in brain metastases as predictive factor of response to systemic therapy in non-small cell lung cancer patients: a preliminary study. Ann Transl Med 9(8):648. 10.21037/atm-20-6497PMID: 33987346; PMCID: PMC8106019 33987346 10.21037/atm-20-6497 PMC8106019

[30] Lin NU, Lee EQ, Aoyama H, Barani IJ, Barboriak DP, Baumert BG, Bendszus M, Brown PD, Camidge DR, Chang SM, Dancey J, de Vries EG, Gaspar LE, Harris GJ, Hodi FS, Kalkanis SN, Linskey ME, Macdonald DR, Margolin K, Mehta MP, Schiff D, Soffietti R, Suh JH, van den Bent MJ, Vogelbaum MA, Wen PY (2015) Response Assessment in Neuro-Oncology (RANO) group. Response assessment criteria for brain metastases: proposal from the RANO group. Lancet Oncol.;16(6):e270-8. doi: 10.1016/S1470-2045(15)70057-4. Epub 2015 May 27. PMID: 26065612 10.1016/S1470-2045(15)70057-4 26065612

[31] Talbot D, Massamba VK (2019) A descriptive review of variable selection methods in four epidemiologic journals: there is still room for improvement. Eur J Epidemiol.;34(8):725–730. 10.1007/s10654-019-00529-y. Epub 2019 Jun 3. PMID: 31161279 10.1007/s10654-019-00529-y 31161279

[32] Singh K, Saxena S, Khosla AA, McDermott MW, Kotecha RR, Ahluwalia MS (2022) Update on the management of Brain Metastasis. Neurotherapeutics 19(6):1772–1781. 10.1007/s13311-022-01312-wEpub 2022 Nov 23. PMID: 36422836; PMCID: PMC9723062 36422836 10.1007/s13311-022-01312-w PMC9723062

[33] Harris AL (2002) Hypoxia–a key regulatory factor in tumour growth. Nat Rev Cancer.;2(1):38–47. 10.1038/nrc704. PMID: 11902584 10.1038/nrc704 11902584

[34] Tang M, Bolderson E, O’Byrne KJ, Richard DJ (2021) Tumor Hypoxia drives genomic instability. Front Cell Dev Biol 9:626229. 10.3389/fcell.2021.626229PMID: 33796526; PMCID: PMC8007910 33796526 10.3389/fcell.2021.626229 PMC8007910

[35] Zhu P, He F, Hou Y, Tu G, Li Q, Jin T, Zeng H, Qin Y, Wan X, Qiao Y, Qiu Y, Teng Y, Liu M (2021) A novel hypoxic long noncoding RNA KB-1980E6.3 maintains breast cancer stem cell stemness via interacting with IGF2BP1 to facilitate c-Myc mRNA stability. Oncogene 40(9):1609–1627. 10.1038/s41388-020-01638-9Epub 2021 Jan 19. PMID: 33469161; PMCID: PMC7932928 33469161 10.1038/s41388-020-01638-9 PMC7932928

[36] Lendeckel U, Wolke C (2022) Redox-Regulation in Cancer Stem cells. Biomedicines 10(10):2413. 10.3390/biomedicines10102413PMID: 36289675; PMCID: PMC9598867 36289675 10.3390/biomedicines10102413 PMC9598867

[37] Viallard C, Larrivée B (2017) Tumor angiogenesis and vascular normalization: alternative therapeutic targets. Angiogenesis 20(4):409–426. 10.1007/s10456-017-9562-9Epub 2017 Jun 28. PMID: 28660302 28660302 10.1007/s10456-017-9562-9

[38] Zheng R, Li F, Li F, Gong A (2021) Targeting tumor vascularization: promising strategies for vascular normalization. J Cancer Res Clin Oncol 147(9):2489–2505. 10.1007/s00432-021-03701-8Epub 2021 Jun 19. PMID: 34148156 34148156 10.1007/s00432-021-03701-8 PMC11802031

[39] Choi Y, Jung K (2023) Normalization of the tumor microenvironment by harnessing vascular and immune modulation to achieve enhanced cancer therapy. Exp Mol Med 55(11):2308–2319. 10.1038/s12276-023-01114-wEpub 2023 Nov 1. PMID: 37907742; PMCID: PMC10689787 37907742 10.1038/s12276-023-01114-w PMC10689787

