# Correction to: Predictive value of perilesional edema volume in melanoma brain metastasis response to stereotactic radiosurgery

**DOI:** 10.1007/s11060-024-04832-x

**Published:** 2024-09-27

**Authors:** Mariya Yavorska, Miriam Tomaciello, Antonio Sciurti, Elisa Cinelli, Giovanni Rubino, Armando Perrella, Alfonso Cerase, Pierpaolo Pastina, Giovanni Luca Gravina, Silvia Arcieri, Maria Antonietta Mazzei, Giuseppe Migliara, Valentina Baccolini, Francesco Marampon, Giuseppe Minniti, Anna Maria Di Giacomo, Paolo Tini

**Affiliations:** 1 Unit of Radiation Oncology, Department of Medicine, Surgery and Neurosciences, University of Siena, Siena, Italy; 2 Radiation Oncology, Policlinico Umberto I, Department of Radiological, Oncological and Pathological Sciences, Sapienza University of Rome, Rome, Italy; 3 Department of Public Health and Infectious Diseases, Sapienza University of Rome, Rome, Italy; 4 Unit of Neuroradiology, Azienda Ospedaliero-Universitaria Senes, Siena, Italy; 5 Department of Biotechnological and Applied Clinical Sciences, University of L’Aquila, L’Aquila, Italy; 6 Policlinico Umberto I Hospital, Viale del Policlinico, Rome, 00161 Italy; 7 Unit of Diagnostic Imaging, Department of Medicine, Surgery and Neurosciences, University of Siena, Siena, Italy; 8 IRCSS Neuromed, Pozzilli, Italy; 9 Center for Immuno-Oncology, Department of Medicine, Surgery and Neurosciences, University of Siena, Siena, Italy

**Journal of Neuro-Oncology**

10.1007/s11060-024-04818-9

In this article the author names Miriam Tomaciello and Antonio Sciurti were incorrectly written as Miriam Tomiciello and Sciurti Antonio, resp.

The original article has been corrected.

